# Comparative analysis of direct coupling and MPPT control in standalone PV systems for solar energy optimization to meet sustainable building energy demands

**DOI:** 10.1038/s41598-024-72606-6

**Published:** 2024-10-02

**Authors:** Chandrasekharan Nataraj, G. Karthikeyan, G. Jaya Bharathi, Shankar Duraikannan

**Affiliations:** 1 School of Engineering, Asia Pacific University of Technology and Innovation, Technology Park Malaysia, 57000 Kuala Lumpur, Malaysia; 2 Anjalai Ammal Mahalingam Engineering College, Chennai, India; 3 SME- Electrical, L&T Edutech, Chennai, India

**Keywords:** Energy science and technology, Engineering, Optics and photonics

## Abstract

Solar energy, a prominent renewable source, has reached an installed capacity of 71.78 GW in India. This research explored the load demands of the computer center at an engineering college in Tanjore, Tamil Nadu, India. The computer center at the engineering college has an annual energy requirement of 260,552 kWh/Year. Consequently, the research focused on the planning and implementation of a standalone photovoltaic (SAPV) system, assessing it against the institution's total annual energy consumption. The performance ratio and losses of the SAPV system with both direct coupling and an MPPT charge controller was compared. This comparative analysis aimed to evaluate the efficacy of two solar photovoltaic control methods—SAPV direct coupling and Maximum Power Point Tracking control—in optimizing energy harvesting from solar panels.

## Introduction

A nation's sustainable development is intricately tied to its energy resources. Green energy, often synonymous with renewable energy, is known for its superior environmental benefits. Historically, around 11% of global energy production comes from renewables, a trend also observed in the United States. Despite ongoing fossil fuel use, the oil crises of the 1970s spurred increased investments in alternative energy. Governments globally have implemented incentives and standards to meet rising public demand for non-fossil fuel energy, driven by climate change concerns^1^.

Solar energy, particularly in countries like India, has gained prominence, boasting an installed renewable energy capacity of 87,669 MW as of June 2020, with solar contributing significantly at 34,811.78 MW. The cost of solar energy has sharply declined to $3.13 per kilowatt-hour, down from $20 per kilowatt-hour over the past 5 years. Solar PV systems play a pivotal role in combating global warming, requiring energy storage solutions, location-specific design parameters, and diverse applications beyond electricity generation. Optimizing solar PV performance necessitates coolants and advanced modelling tools, offering promising avenues for enhancing efficiency and advancing sustainable energy development^2,3^.

Solar PV systems are widely adopted for harnessing solar energy, playing a crucial role in addressing global warming and meeting climate targets^4,5^. These systems convert solar energy into electricity, with a key consideration being the storage of generated electricity for use during periods without sunlight^6,7^. Rooftop systems significantly reduce peak energy costs for residential and MSME sectors^8–10^. Important variables include the performance ratio, system losses, and solar yield, influenced by factors like the environment, mounting technique, and electrical construction. Performance on hot days can be improved using coolants to prevent solar PV cell modules from overheating^11–13^.

In recent years, more efficient control methods have been developed, either solo or in conjunction with other renewable energy sources such as wind, to offer dependable, cost-effective, and high-quality power from the sun and the wind^14–16^. Real-world challenges and uncertainties that may arise in integrating diverse renewable energy sources within a standalone microgrid while designing, developing, operating, and maintaining rooftop PV systems require ideal location-specific process parameters^17^. Subsequently, many researchers have developed standalone and eco-friendly micro-grids for solar systems using cutting-edge optimisation algorithms such as fuzzy logic, metaheuristics such as the wild horse optimizer, gravitational search algorithm, and genetic algorithm^18,19^.

The optimization of SPV systems under partial shading (PS) conditions is crucial for enhancing cleaner energy production. When mounted solar panels are used to gather energy from the sun, they do not make the best use of the resource since the sun's rays do not constantly remain perpendicular to the axis of the solar cells throughout the day, reducing production^20–22^. To address this angle and alignment issues, the option of using solar trackers is reported in the existing research aligned with the solar energy harvesting strategies^23,24^. Furthermore, most of the systems are significantly built in a very effective way by adopting maximum power point tracking technique with the support of artificial neural networks^25,26^. Photovoltaic (PV) and wind energy have become more popular due to their quantity of renewable energy supplies. Of the variety of demands, domestic electrification significantly required a backup energy source that can ensure a consistent supply of electricity^8,27^. As a result, the renewable sector still has opportunity for expansion, and providing optimized solutions for the different issues addressed in this solar energy harvester is critical. More research is needed to identify realistic approaches to improve the performance of energy harvesters.

This paper focuses on evaluating the potential of solar energy, establishing the minimum load for operation at a specific location during power outages, strategizing the configuration of PV systems, and computing performance ratios and losses**.**

The findings of this research will be widely used to simulate and analyze the performance of solar photovoltaic systems. The system's uniqueness resides in the fact that SAPV is designed using real-time irradiation data for a specific geographic region. The uncertainty of the irradiations is considered to the greatest extent possible when generating to meet load demand. This ensures that the proposed solution is directly appropriate to the energy requirements of the specific area. Even if the load requirement increases in the future, the same system may be used with minor alterations. It reduces the stress of focusing too much on the new system development. This research intends to significantly reduce the amount of time spent working on every upgrade.

However, the performance of energy harvester systems and its subcomponents, highly depends on the conditions of externally available energy source. The system's drawback is that it relies only on solar energy, making it weather dependent. Furthermore, while including batteries for energy storage is useful, it raises the initial cost, creating a financial barrier to implementation.

## System Model

**Fig. 1:**
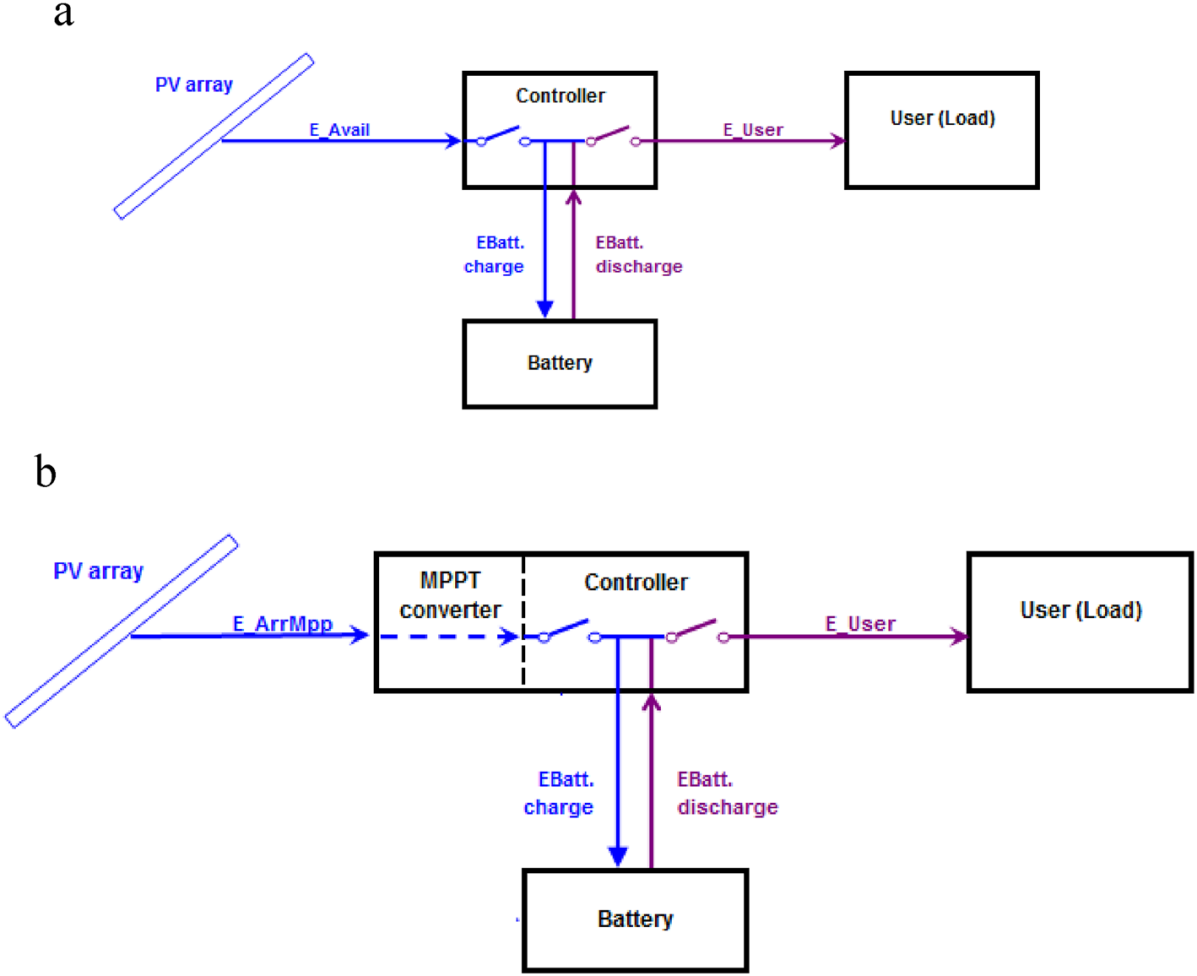
(**a**) Layout of direct coupled SAPV system. (**b**) Layout of SAPV system with MPPT Controller.

This research EXAMINED the impact of solar power plant size on losses and performance ratios in a standalone photovoltaic system. Both charge controller and MPPT techniques were analysed using PVsyst simulations to investigate losses across diverse parameters and assess the performance ratio. The primary focus was on energy metrics, solar resource utilization, and the overall implications of performance ratios and losses within the plant. Figures 1a and 1b illustrated the configuration of a standalone PV system for direct coupling and integration with an MPPT charge controller. The intelligent MPPT charge controller optimizes the system's efficiency by continuously monitoring the electrical output from the solar panels, ensuring maximum power point operation under varying environmental conditions. This process enhanced the charging efficiency of the associated battery bank, resulting in improved total energy harvest, with the batteries functioning autonomously for power storage.

### Standalone PV system design

Standalone rooftop systems, independent of the power grid, operates on batteries and consist of solar modules, a controller, and an inverter^1,28^. The solar modules, attached to a mounting framework, generate DC electricity, which is then directed through the charge controller to charge the battery. The controller serves the dual functions of battery charging and preventing overcharging, including inhibiting reverse current flow from batteries to solar panels at night. The stored energy in the battery can be utilized throughout the day and night, with the inverter converting it for immediate use whenever required.

**Fig. 2:**
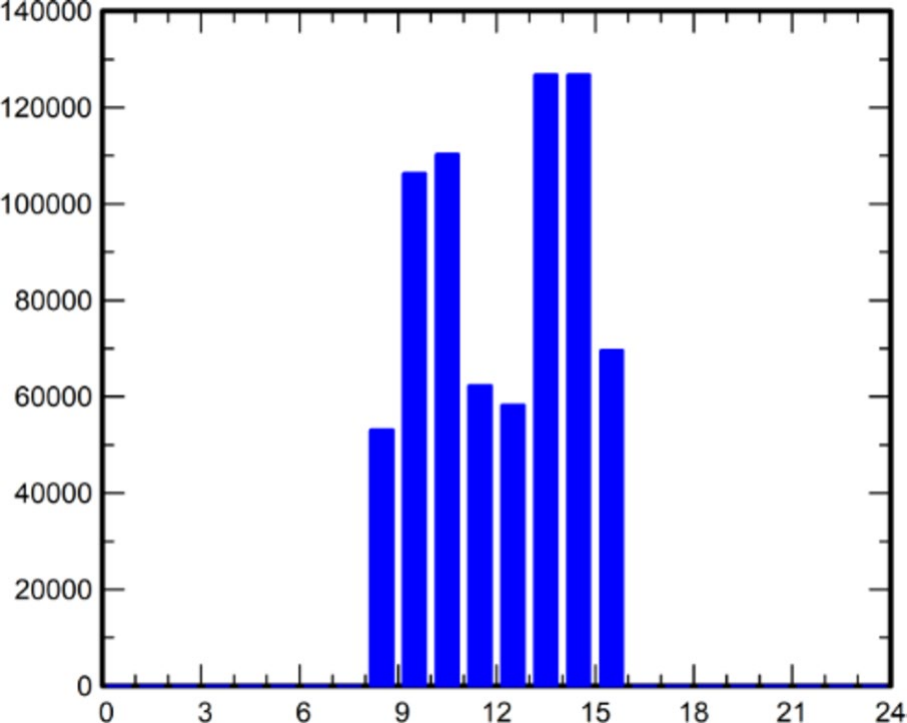
Daily load profile.

Daily load patterns can vary seasonally in some areas, with higher cooling demand during hot summer afternoons leading to a more prominent afternoon peak. Utilities and energy suppliers use daily load profiles to enhance power generation and distribution strategies. Consumers can leverage this information to optimize the design of solar PV arrays and batteries, potentially reducing energy costs. Figure 2 displays the daily load profile of an engineering college computer block, indicating hourly load distribution from 8 a.m. to 4 p.m. The load is assumed to remain constant throughout the year, with an average daily energy consumption of 714 kWh.

The PV panels were designed based on the daily load profile, utilizing PVsyst software. The solar panel system incorporates 1110 PV modules from HBL Power Systems Ltd, each rated at 250 Watts peak (Wp). These modules were organized into 370 strings, with each string consisting of 3 modules in series. The overall system has a total nominal capacity of 278 kWp, but its maximum power under standard conditions is 252 kWp. Table 1 presents the panel array system design specifications.

**Table 1:** Panel array system design specification.

PV module manufacturer	HBL Power Systems Ltd
Unit Nom. Power	250 Wp
Number of PV modules	1110 units
Nominal (STC)	278 kWp
Modules	370 Strings × 3 In series
Pmpp	252 kWp
U mpp	94 V
I mpp	2679 A

The battery system was configured to sustain the load autonomously for two days. To determine the necessary capacity, an initial estimate of the target system's daily energy consumption is made, accounting for all essential devices and systems. This estimation included an evaluation of potential energy losses during charging and discharging. The required battery capacity was then calculated by multiplying the daily energy usage by the two-day autonomy time. Refer to Table 2 for the detailed battery specifications of the SAPV system.

**Table 2:** Battery design specification.

Battery manufacturer	Rolls
Technology	Lead-acid sealed, plates
No of units	94 in parallel × 6 in series
Discharging min SoC	20%
Stored energy	1602.7 kWh
Nominal capacity	27,824 Ah (C10)

The SAPV system is structured around a 1000 W and 24 V universal controller, which allows for maximum charging and discharging currents ranging from 32 to 20 A. Table 3 displays the MPPT controller parameter generated by the Generic manufacturer, which has a nominal battery voltage of 72 V. The highest input current is 4237 A, while the maximum output current is 1761 A.

**Table 3:** MPPT controller parameters.

Manufacturer	Generic
Nominal battery voltage	72 V
Maximum input current	4237 A
Maximum output current	1761 A

### Geographical location and panel orientation

**Fig. 3:**
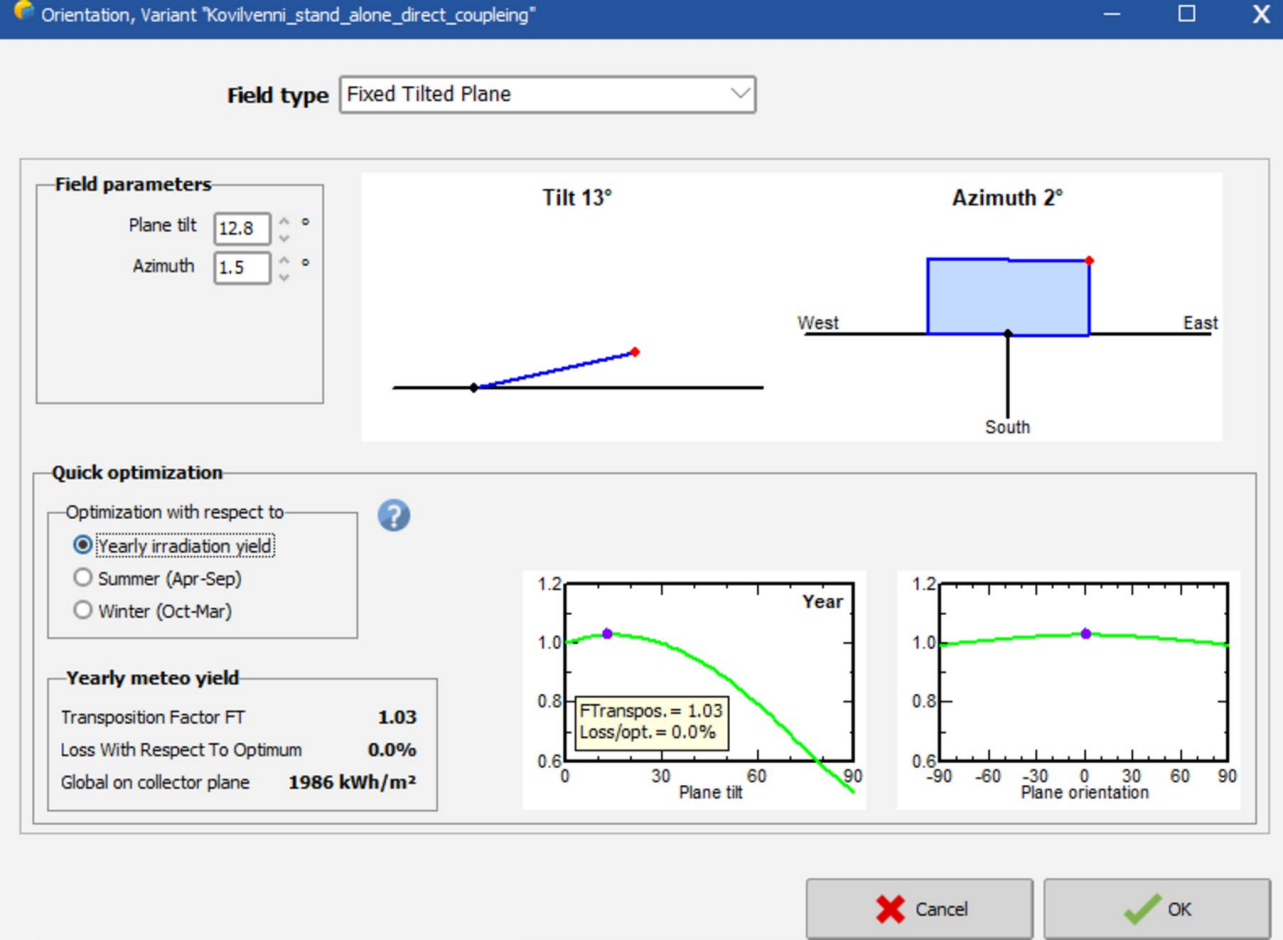
Panel orientation for the proposed site.

The site was situated at 10.7811° N Latitude and 79.3631° E Longitude. Illustrated in Fig. 3, the field featured a structure comprising fixed tilted panels at 12.8 degrees and an azimuth angle of 1.5 for the chosen field. The panel collected an energy amount of 1986 kWh/m2, and the optimization process covered the entire year with the objective of minimizing losses to zero percent.

## Simulation result and discussion

The PVsyst program is used to generate the simulation results. The simulation results for direct connection and the MPPT charge controller are compared. SAPV modelling using a charge controller and MPPT is investigated using various factors such as performance ratio, losses, unused energy, and solar fraction as listed in Table 4.

**Table 4:** SAPV energy metrics and battery performance.

Specification	Direct coupled SAPV	SAPV with MPPT controller
Available energy	346,264 kWh/year	**395,616 kWh/year**
Used energy	258,002 kWh/year	**260,552 kWh/year**
Excess (unused)	69,929 kWh/year	**113,284 kWh/year**
Missing energy	2550 kWh/year	**0 kWh/year**
Specific production	1248 kWh/kWp/year	1426 kWh/kWp/year
Performance ratio (PR)	50.57%	51.07%
Solar fraction (SF)	99.02%	100%
Battery aging (state of wear)		
Cycles SoW	97.8%	97.4%
Static SoW	89.2%	89.2%
Battery lifetime	9.2 years	9.2 years

Significant values are in [bold]

For the period, the available energy resources are 346,264 kilowatt-hours per year (kWh/year). 258,002 kilowatt-hours per year (kWh/year) of this available energy have been consuming, leaving a surplus of energy 69,929 kWh/year. However, there is a missing energy of 2550 kilowatt-hours per year (kWh/year) when the SAPV is directly coupled.

Table 5 details monthly energy measurements and an annual summary. In January, the available energy is 30,725 kWh, with 22,129 kWh effectively utilized, 6897 kWh remaining unused, and no energy losses. This trend, inclusive of solar radiation and consumption factors, remains consistent, maintaining a solar fraction (SolFrac) of 1.000 throughout most of the year. However, deficits in available energy during November and December lead to missing energy (E_Miss) and lower solar fractions of 0.906 and 0.976, respectively.

**Table 5:** Annual energy Production and consumption of Direct coupled SAPV.

	GlobHor kWh/m^2^	GlobEff kWh/m^2^	E_Avail kWh	EUnused kWh	E_Miss kWh	E_User kWh	E_Load kWh	SolFrac ratio
January	146.6	158.5	30,725	6897	0	22,129	22,129	1
February	146	152.7	29,744	7756	0	19,988	19,988	1
March	178.5	178.6	34,818	10,543	0	22,129	22,129	1
April	176.3	168.4	32,783	9388	0	21,415	21,415	1
May	170.5	156.5	30,421	6871	0	22,129	22,129	1
June	171	153.4	29,829	7019	0	21,415	21,415	1
July	161.3	146.9	28,534	5086	0	22,129	22,129	1
August	159.2	150	29,161	5290	0	22,129	22,129	1
September	152.6	149.3	28,916	6308	0	21,415	21,415	1
October	127.3	128.6	24,514	1288	0	22,129	22,129	1
November	111.5	116.7	22,085	931	2023	19,392	21,415	0.906
December	119.8	128.4	24,733	2552	526	21,603	22,129	0.976
Year	1820.6	1788	346,264	69,929	2550	258,002	260,552	0.990

GlobHor: Horizontal global irradiation. GlobEff: Effective global, corr. for IAM and shadings. E_Avail: Available Solar Energy. EUnused: Unused energy (full battery) . E_Miss: Missing energy. E_User: Energy supplied to the user. E_Load: Energy need of the user (Load) . SolFrac: Solar fraction (EUsed/ELoad).

The annual summary underscores that 69,929 kWh of the total available energy (346,264 kWh) is unused, constituting waste. In contrast, a substantial 260,552 kWh, equivalent to 99% of the available energy, is effectively consumed.

**Fig. 4:**
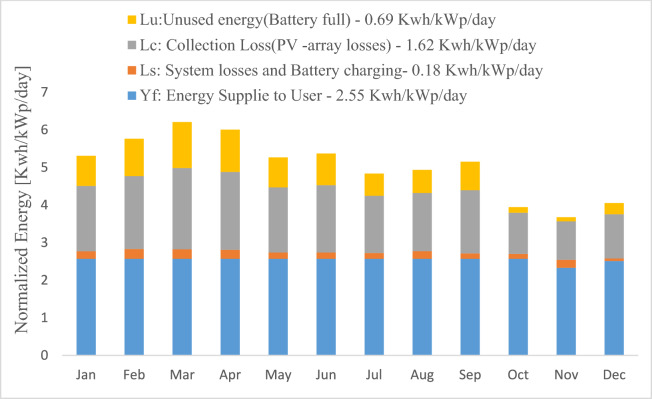
Normalized production/installed kWp of Direct coupled SAPV system.

Figure 4 provides crucial metrics for evaluating a solar photovoltaic (SAPV) system’s efficiency, including performance ratios and normalized energy production per kilowatt-peak of installed capacity. Specific losses, such as Lu (0.69 kWh/kWp/day) for unused energy due to a fully charged battery, Lc (1.62 kWh/kWp/day) for energy losses from PV-array inefficiencies, and Ls (0.18 kWh/kWp/day) for losses tied to system performance and battery charging, are considered. The total normalized energy production, factoring in these components, is 2.55 kWh/kWp/day. However, a detailed monthly breakdown of these metrics from January to November, crucial for a thorough assessment of the system's performance, is not provided in the current data.

**Fig. 5:**
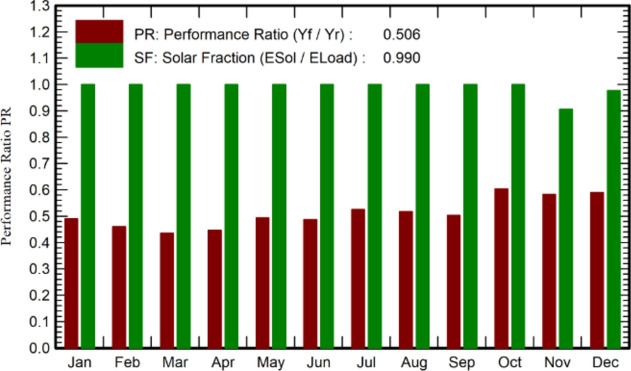
Performance ratio of the direct coupled SAPV system.

Figure 5 displayed two significant performance metrics: the Performance Ratio (PR) at 0.506, assessing the system's efficiency in converting solar energy into electricity by comparing actual and expected energy production, and the Solar Fraction (SF) at 0.990, indicating that solar energy covered 99% of the total energy demand. The absence of specific monthly figures from January to November highlighted the need for this monthly data to obtain a thorough view of the system’s annual performance.

**Fig. 6:**
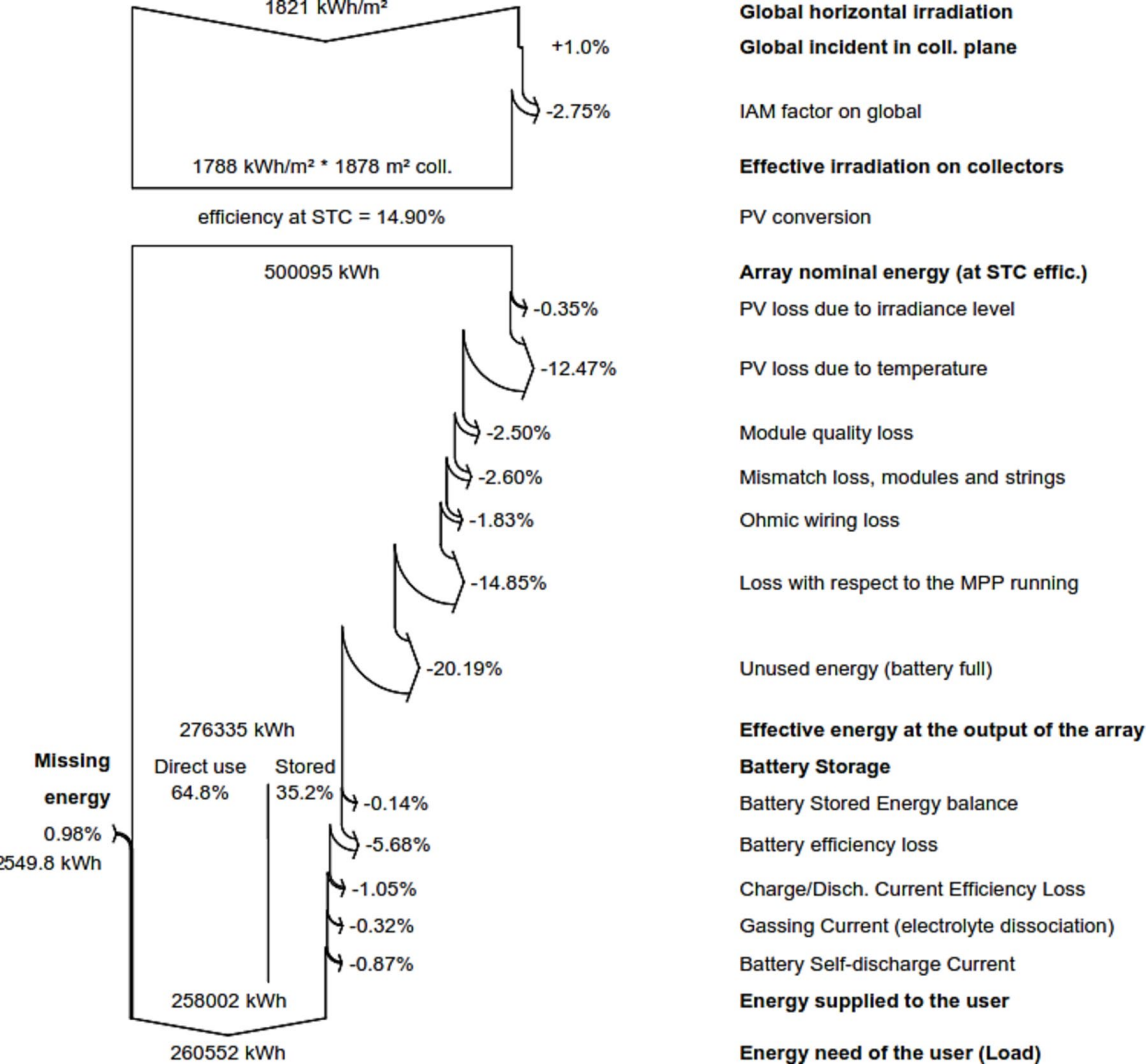
Detailed loss structure of Directed coupled SAPV system.

Figure 6 detailed metrics related to a photovoltaic system, covering energy generation, conversion, storage, and losses. It included global horizontal irradiation, collector plane incident energy, and the IAM factor, resulting in an effective irradiation of 1788 kWh/m^2^ on an 1878 m^2^ collector.

The dataset explored the photovoltaic system's performance, highlighting a 14.90% efficiency under standard testing conditions and a total array nominal energy of 500,095 kWh. Various losses were considered, such as irradiance and temperature losses, module quality, mismatch loss, ohmic wiring, and efficiency reductions at the maximum power point. The effective energy output at the array was evaluated at 294,530 kWh, accounting for converter losses during operation, resulting in a total of 281,228 kWh. Additionally, the data examined battery storage efficiency and losses, revealing that 61% of the energy was directly utilized, with the remaining 39% being stored. A minor 0.07% balance was noted. Battery efficiency was impacted by 6.15%, resulting in a total battery efficiency loss of 1.15%. In summary, the data showed that 260,552 kWh of energy was supplied to meet needs of the users.

**Fig. 7:**
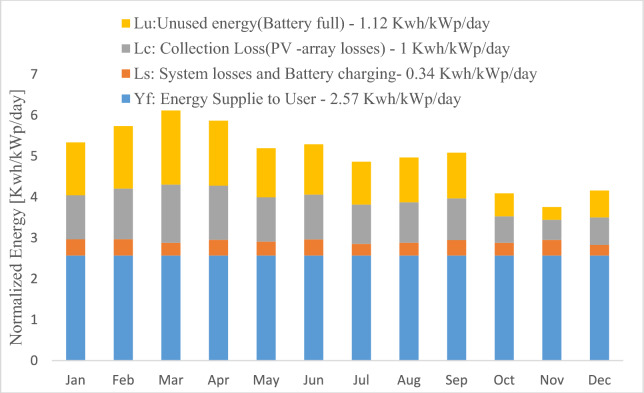
Normalized production/installed kWp SAPV system with MPPT controller.

Figure 7 presented the normalized energy output per installed kilowatt-peak (kWp) in a photovoltaic (PV) system, detailing key aspects: normalized energy production per kilowatt-peak of installed capacity, 1.12 kWh/kWp per day of unused energy due to a fully charged battery (Lu), 1 kWh/kWp per day representing losses linked to PV-array inefficiencies (Lc), and 0.34 kWh/kWp per day for system losses and energy utilized for battery charging (Ls). The data also indicated the effective energy supplied to meet user requirements, quantifying at 2.57 kWh/kWp per day. However, specific numerical values for each month were not provided, emphasizing the necessity of including monthly data for a comprehensive evaluation of the system's annual performance.

**Fig. 8:**
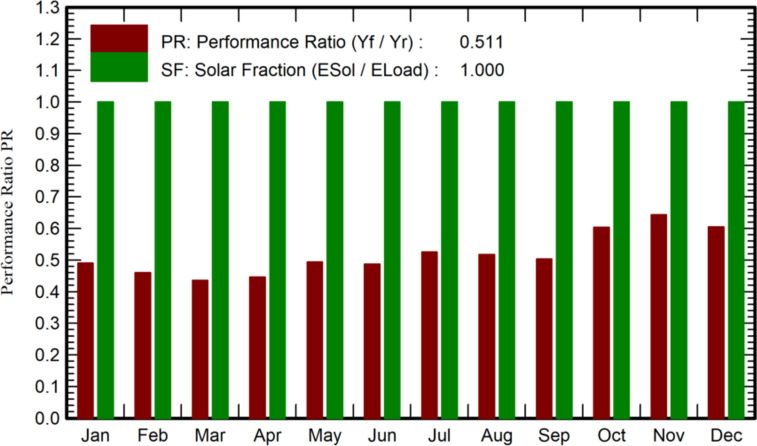
Performance ratio of SAPV system with MPPT controller.

Figure 8 highlighted key performance metrics: a Solar Fraction (SF) at 1.000, indicating complete fulfilment of energy demand through solar power, showcasing high efficiency; and a Performance Ratio (PR) with a value of 0.51, serving as an indicator of the system's effectiveness by comparing actual energy production to expected levels. These metrics provided essential insights into the system's operation and its capacity to generate power from solar energy while meeting energy demand.

Table 6 displayed various energy metrics on a monthly and annual basis, including values such as Global Horizontal Irradiation (GlobHor), Effective Global Solar Energy (GlobEff), Available Solar Energy (E_Avail), Unused Energy (EUnused), Missing Energy (E_Miss), Energy Supplied to the User (E_User), Energy Need of the User (E_Load), and the Solar Fraction (SolFrac). Throughout the year, the system consistently maintained a Solar Fraction of 1.000, indicating the efficient fulfilment of 100% of the total energy demand. Notably, there was no energy loss (E_Miss), with 113,284 kWh remaining unused (EUnused) out of the 395,616-kWh available. Moreover, the system successfully delivered 260,552 kWh of energy (E_User) to meet the user's energy requirements, demonstrating highly effective energy utilization.

**Table 6:** Annual energy Production and consumption of SAPV with MPPT.

	GlobHor kWh/m^2^	GlobEff kWh/m^2^	E_Avail kWh	EUnused kWh	E_Miss kWh	E_User kWh	E_Load kWh	SolFrac ratio
January	146.6	158.5	35,570	11,120	0	22,129	22,129	1
February	146	152.7	34,042	11,889	0	19,988	19,988	1
March	178.5	178.6	39,372	15,582	0	22,129	22,129	1
April	176.3	168.4	36,806	13,237	0	21,415	21,415	1
May	170.5	156.5	34,276	10,340	0	22,129	22,129	1
June	171	153.4	33,876	10,256	0	21,415	21,415	1
July	161.3	146.9	32,488	9023	0	22,129	22,129	1
August	159.2	150	33,144	9434	0	22,129	22,129	1
September	152.6	149.3	32,711	9312	0	21,415	21,415	1
October	127.3	128.6	28,429	4823	0	22,129	22,129	1
November	111.5	116.7	26,014	2613	0	21,415	21,415	1
December	119.8	128.4	28,887	5657	0	22,129	22,129	1
Year	1820.6	1788.0	395,616	113,284	0	260,552	260,552	1

**Fig. 9:**
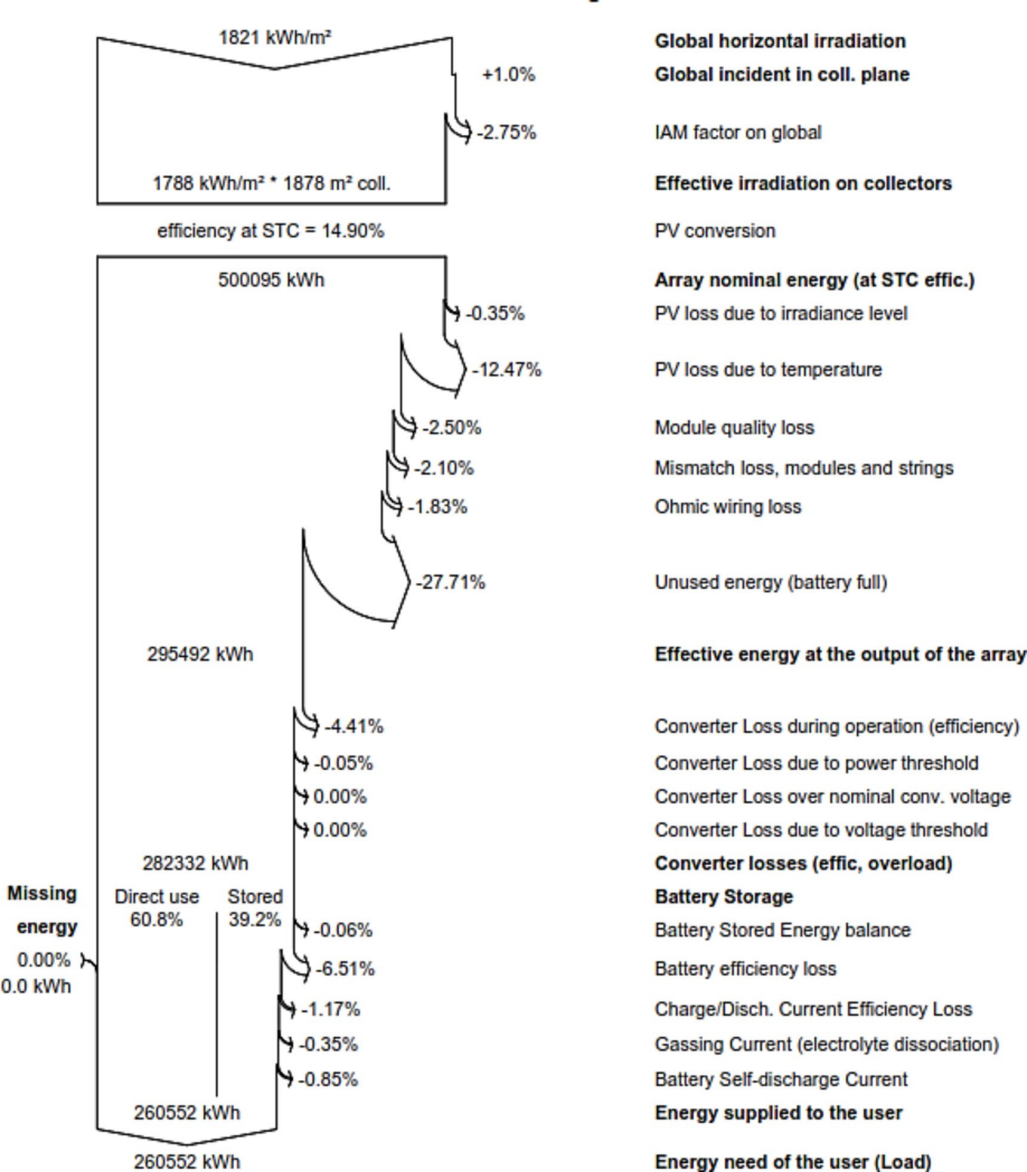
Detailed loss structure of SAPV system with MPPT controller.

Figure 9 provided detailed data on energy production, conversion, and storage within a photovoltaic system, offering precise numerical values for each component. The system's efficiency under Standard Testing Conditions (STC) is recorded at 14.90%, generating an Array Nominal Energy of 500,095 kWh. Various losses, including those due to irradiance level, temperature, module quality, mismatch losses, and ohmic wiring, resulted in a significant 27.71% of unutilized energy when the battery was at full capacity. The Effective Energy output at the array, accounting for converter losses, quantified at 295,492 kWh. Battery storage reflected a 60.8% direct utilization of generated energy, with 39.2% being stored. Battery efficiency experienced a 6.51% loss, comprising reductions due to charge/discharge current efficiency, gassing current, and battery self-discharge current. In summary, the energy supplied to meet user needs equals 260,552 kWh, aligning with the user's energy demand (Load). Notably, no missing energy was reported. This dataset provided a comprehensive and quantitative insight into the intricate energy dynamics and losses within the system.

**Fig. 10:**
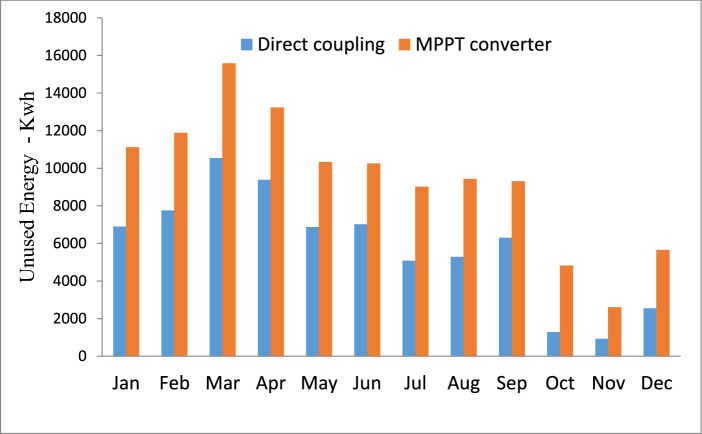
Energy Unused Comparison: Direct Coupling vs. MPPT Converter.

Figure 10 demonstrated that adopting an MPPT converter resulted in an increase of unused energy to 113,286 kWh annually compared to 69,929 kWh with direct connection. The use of an MPPT converter capable of exporting power to the grid leads to a 62% enhancement in power harvesting from the same system. Additionally, the MPPT Controller eliminates the loss of 2550 kWh of energy in the months of November and December.

As a result of the findings, the suggested research focuses on freestanding photovoltaic systems. The proposed SAPV system is a practical and unique strategy that uses real-time data for a specific geographic region, assures adaptation to future demands, and contributes to the broader goal of sustainability and climate resilience in renewable energy systems.

## Conclusion

The paper assessed the load requirements for an engineering college's computer block, designing a standalone system based on daily load profiles. Utilizing PVsyst software, the analysis included performance ratio and losses. The annual energy requirement was 260,552 kWh, and with direct coupling, 258,002 kWh was supplied, slightly below the load. After implementing MPPT, the supplied energy matched the full demand. The MPPT system improved the performance ratio from 50.57 to 51.07%, achieving a solar fraction of 100%. With 62% more energy harvested, 113,286 kWh/year can be exported to the grid. The study informs on-grid solar PV system development, emphasizing economic analysis. PV framework efficiency depends on technology and manufacturing, with uncertainties in meteorological data, PV module models, and manufacturing details. Rooftop solar panels tailored to load requirements enable self-sufficiency for domestic or small-scale industrial use.

By taking these characteristics into account, future research can help to design renewable energy systems that are more linked, resilient, and efficient, using synergies between different renewable sources. This comprehensive plan strives to create coherent systems that not only meet energy needs but also contribute to larger sustainability and climate resilience goals.

## Data Availability

All data generated or analysed during this study is included in the main content of this publication. The OneDrive link is given below to access both figures and tables used in the manuscript. https://cloudmails-my.sharepoint.com/:u:/g/personal/chandrasekharan_apu_edu_my/EZCYvVDlIoNFsFh2xUWxH9ABiHIjJly4wkn4pF8maAHN1A?e = TX9Wle.
